# Reducing hospital admissions in remote Australia through the establishment of a palliative and chronic disease respite facility

**DOI:** 10.1186/s12904-017-0247-3

**Published:** 2017-11-21

**Authors:** Timothy A. Carey, Mick Arundell, Kellie Schouten, John S. Humphreys, Fred Miegel, Simon Murphy, John Wakerman

**Affiliations:** 1 Centre for Remote Health, a centre of Flinders University, PO Box 4066, Alice Springs, NT 0871 Australia; 2 Northern Territory Department of Health, Darwin, Australia; 3 Monash University School of Rural Health, Melbourne, Australia

**Keywords:** Palliative care, Respite care, Indigenous, Chronic disease, National weighted activity unit

## Abstract

**Background:**

There are limited respite services for palliative care patients and their families in the Northern Territory (NT). The high prevalence of complex chronic diseases, limited access to primary care services, and the poor living situations of many Aboriginal and Torres Strait Islander Australians result in high hospitalisation rates and pressure on tertiary health services. Palliative Care NT identified a need for a flexible, community based, culturally appropriate respite service in Alice Springs. It was of particular interest to assess the impact of the respite service on the extent to which hospital resources were accessed by this population of patients.

**Methods:**

Respite service use and hospital use data were collected over two time periods: the 12 months prior to the establishment of the service; and the first 10 months of the operation of the service. The financial implications of the facility were assessed in terms of the National Weighted Activity Unit (NWAU). Of primary interest in this study was the impact of the respite service on admissions to the Emergency Department (ED), to the Wards, and to the Intensive Care Unit (ICU). The amount of ventilator hours consumed was also of interest.

**Results:**

Overall, there was a mean cost saving of $1882.50 per episode for hospital admissions with a reduction in: hospital admissions; mean length of stay; Intensive Care Unit (ICU) hours; and ventilator hours.

**Conclusions:**

The establishment of the respite service has met an important and unmet need in Alice Springs: provision of respite where none has existed before. The service did assist with savings to the health department which could contribute to the cost of the facility over time. Two features of the respite facility that may have contributed to the savings generated were the enhanced coordination of care for patients with complex chronic diseases, as well as improved medication compliance and symptom management.

## Background

Palliative care in the Northern Territory (NT) operates primarily as a consultancy based service supporting the work of acute and primary care providers and community nurses providing direct care to patients residing in their homes. In central Australia, 66% of the palliative care caseload comprises Aboriginal and Torres Strait Islander people. Due to the high Aboriginal and Torres Strait Islander population in the NT, their poorer health status, and higher rates of premature mortality and chronic disease, palliative care is used differently compared with other Australian jurisdictions. Whereas in mainstream Australian palliative care, cancer patients are the biggest diagnostic group to access palliative care services, making up about 60% of palliative care usage, followed by cardiovascular disease and respiratory disease patients (both 8%), chronic disease constitutes a larger proportion of the palliative care workload in the NT than cancer [1].

The NT has the highest burden of chronic disease in Australia compared with other states and territories. NT Aboriginal and Torres Strait Islander people suffer from chronic disease at a rate of 2.5 times the rate for other Australians [2]. Using the Disability Adjusted Life Year measure, Zhao et al. [2] estimated that between 30th December 1985 and 1st Jan 1994, 127,900 years of life were lost as a result of chronic disease, and of these, almost half were attributable to Aboriginal and Torres Strait Islander people who make up only one third of the NT population.

High rates of chronic disease in the NT have a significant impact on hospitalisation. Between 1998 and 99 and 2005–06, 15.6% of the total NT hospitalisations were avoidable and Aboriginal and Torres Strait Islander people accounted for 61% of these avoidable hospitalisations. The avoidable hospitalisation rates in the NT exceed the Australian rates for almost all conditions and are largely attributable to chronic disease [3].

In the NT, currently 80% of people die in institutions and Aboriginal and Torres Strait Islander people are more likely to die in institutions compared to other people. Dying at home, however, is a strong preference for people generally [4] and Aboriginal and Torres Strait Islander people in particular [5]. Hospital deaths also result in an increased financial burden on the health system [6] and a potentially poorer quality of death [7]. Respite facilities have a role in supporting people with their wishes to die at home by providing community based professional palliative care support. Recent literature reviews of palliative care services indicate that it is important for services to attend to patient’s psychosocial needs as well as their physical needs [8] and that it is also essential that staff are well trained with clearly defined roles [4]. The respite service that was established provided a facility for patients to meet and socialise with each other and with staff. The staff of the service were well trained health professionals with a special interest in palliative care.

### The current study

The establishment of a day respite facility in Alice Springs provided an opportunity to examine the benefits to patients and carers in terms of an improved quality of life and also to hospitals in terms of reduced admissions and financial savings. Improvements in quality of life for both patients and carers were demonstrated through the qualitative study [9]. The current paper reports on the financial impact on the hospital of the respite service. In particular, it was of interest to establish if there had been any changes in hospital admissions and ventilator hours after the establishment of the respite service. Specifically, the research question that guided this study was: What happens to hospital admissions and ventilator hour usage following the introduction of a day respite service? Results related to the impact on patients and carers as well as the clinical effectiveness of the service are beyond the scope of the current study but have been discussed extensively in the qualitative study accompanying this research [9].

## Method

### The respite facility

Through Commonwealth funding, a suburban house was purchased and refurbished as the respite facility. The funding also enabled operation of the facility. The respite facility was staffed by nursing staff which enabled patients attending the facility to have some of their medical needs attended to. Broad referral criteria were established with relevant agencies in Alice Springs and, once referred, patients could attend the facility during weekly working hours. While at the facility, participants could engage in various unstructured leisure and social activities such as cooking, watching television, and socialising with staff and other patients. A qualitative study of the impact of the facility on patients and carers indicated that the facility had a substantial positive impact on both patients’ and carers’ quality of life and that spending time at the facility was highly valued [9]. During the study period, 23 people were referred to the service by seven different agencies including the Alice Springs Hospital, a Health Department Palliative Care Service, an Aboriginal Medical Service, and an Aboriginal Aged Care Service. Of those who were referred, 17 people went on to use the service and 13 people used the service on a regular basis.

### Research design and organisation of data

A pre and post design was used to compare changes in Emergency Department (ED) attendances and hospital admissions following the commencement of the service, as well as the financial implications of these changes in terms of the National Weighted Activity Unit (NWAU). These data were collected from the hospital database (Caresys) from 1 August 2012 to 31 July 2013 and for the 10 subsequent months of operation of the respite facility. The Alice Springs Hospital Clinical Data Analyst extracted and analysed these data.

Patterns of service use were derived from data on the hospital database and documented. Consent to obtain patient data was obtained following standard ethical procedures. All 17 patients using the service consented to their data being used in the study. Demographic data of the people using the service were collected. The following categories of data were collected: gender; Aboriginal and Torres Strait Islander status; locality (e.g., town camp/town house); diseases (including stages e.g., COPD, kidney disease, heart failure, cancer); co-morbidities; remote community of origin; origin of referral; level of family support; and level of independence. Clinical information including diagnostic category was collected from hospital records.

### Hospital attendances and cost savings

ED attendances and hospital admissions are recorded on Alice Springs Hospital’s patient database Caresys. The figures recorded in Caresys were used to determine hospital usage. As a standardised measure, hospital expenditure is calculated by multiplying the National Efficient Price (NEP) Determination by the NWAU [10].

An NWAU is the unit used to express the price weights for all services funded on an activity. Units used in the calculation of an NWAU include (among other things): hospital state; rurality; ICU Days; patient age; postcode; length of stay; psychiatric days; and diagnostic related group. The NEP is a base price calculated by the IHPA as a benchmark to guide governments about the level of funding which would meet the average cost of providing acute care (admitted, emergency, and outpatient) services in public hospitals across Australia. The NEP is based on the projected average cost of a National Weighted Activity Unit (NWAU). The NEP for 2012–13 and 2013–14 was $4808 and $4993 respectively per NWAU. Average hospital service is worth one NWAU.

## Results

As mentioned above, in the evaluation period, a total of 23 referrals were received by the respite service from seven different agencies. Of these 23 referrals, 17 patients went on to use the service and 13 patients continued to use the service on a regular basis. Some referrals were accepted that did not strictly meet the criteria, such as continuing patients at Alice Springs Hospital (patients who were living at the hospital) and disability and aged care patients. A small number of patients were not accepted. Reasons for not accepting patients included; patients requiring one-to-one care, group homogeneity, patient load, and staff capacity.

### Patient demographic profile

#### Gender

Both males (13) and females (4) used the respite service. There were no known reasons for the gender imbalance. Of the total number of referrals received, 17 were men, and 6 were women. Therefore, in the evaluation period, men were approximately three times more likely to be referred to the program than women.

#### Usual residence

Patients using the service lived in a variety of places: approximately 41% were residents of a town camp; approximately 29% owned or rented private accommodation; approximately 17% were continuing patients from Alice Springs Hospital waiting placement in other long term facilities; and approximately 11% were homeless.

#### Age

The mean age for patients attending the respite service was 58, with a range of 37 to 83 years of age.

#### Aboriginal and Torres Strait Islander status

Aboriginal and Torres Strait Islander people made up 76% of the patient load: 13 Aboriginal and Torres Strait Islander people and 4 other people.

#### Disease state

Of those who attended the service, 23% suffered from end stage cancer including bone cancer, lymphoma, bladder cancer, and oesophageal cancer. Also, 64% suffered from chronic diseases including cardiovascular, endocrine, renal, respiratory, gastro intestinal, and diseases of the brain, and 12% were classified as disabled (Acquired Brain Injury). Over half of those people attending the service were recorded as having one or more co-morbidities.

#### Cognitive, functional, and mobility impairment

There was some form of cognitive impairment in 47% of patients, including dementia, acquired brain injury, and unspecified cognitive impairment. Functional or mobility impairment was experienced by 52% of patients.

### Changes in admissions and financial consequences

Following the introduction of the respite service there was an increase in ED attendances, an increase in ED hours and admissions, a slight reduction in admissions to the ward from ED, and a substantial reduction in the average length of stay in hospital (see Table 1).

**Table 1:** Changes in patterns of service use and associated costs

	12 Months Prior to the Service Starting		10 Months After the Service Starting	
	Mean	Median	Mean	Median
Number of ED attendances (per month)	4.00	5.50	28.20	11.00
Total ED hours (per month)	138.50	44.20	376.30	64.90
Number of admissions to ED only (per month)	3.80	1.00	19.10	2.00
Number of admissions to ward (per month)	7.00	3.50	5.80	4.00
Length of Stay (days)^a, b^	64.80	34.00	34.20	17.00
ICU hours (per month)	133.20	0.00	2.00	0.00
Ventilation hours^a, c^	67.30	0.00	0.00	0.00
NWAU dollar value per episode for ED attendances	$643.30	$668.50	$679.80	$593.50
NWAU dollar value per episode for hospital admissions	$6159.40	$1515.30	$4276.90	$1188.10

^a^ Defined according to Australian Institute of Health and Welfare definitions

^b^ http://meteor.aihw.gov.au/content/index.phtml/itemId/269422. Accessed 27 Nov 2016.

^c^ http://meteor.aihw.gov.au/content/index.phtml/itemId/479010. Accessed 27 Nov 2016

In the 12 months prior to the commencement of the respite service, there were 326 admissions to Alice Springs Hospital of the patients participating in the study. Their average length of stay was 64.8 days, the average time in ICU was 133.2 h, and there was an average of 67.3 ventilator hours used. In the 10 months after the commencement of the respite service, there were 276 admissions. In this period the mean length of stay decreased by half, the amount of time spent in the ICU reduced to two hours, and there was no time spent on artificial ventilation (see Table 1). Following the commencement of the respite service, the mean cost per hospital admission episode expressed in NWAUs reduced by $1882.50. A saving of $1882.50 per hospital admission for 276 admissions over the 10-month period of the study amounted to a total savings of $519,570.00.

Both means and medians are provided in Tables 1 and 2 to give a sense of the highly skewed nature of the patient distributions. For example, the median ICU hours both before and after the service started was zero. Similarly the mean NWAU dollar value per episode for ED attendances increased from before to after, however, the median decreased. Clearly, there were a small number of patients responsible for most of the hospital usage.

## Discussion

To address the growing need of palliative and chronic disease patients in a remote Australian town, a day respite service was established. As well as demonstrating that the service had beneficial effects on the quality of life of patients and carers [9] it was also of interest to assess the financial implications of the service. The specific questions being answered in this study were: What happens to hospital admission and ventilator hour usage following the introduction of a day respite service. The results were, in some ways, surprising and provide important preliminary information for policy, practice, and future research. Answers to these questions will be expanded upon below but, in brief, results indicate a *reduction* in ED attendances, an *increase* in ED hours and admissions, and a *reduction* in admissions to the ward from ED including ICU. Furthermore, ventilator hours reduced to zero for the study period.

It appears from the results of this study that not tailoring services to accommodate the complex needs of people in central Australia results in overuse of the Alice Springs Hospital, little opportunity for early improvements in symptom management, and palliative and chronic disease patients “slipping through the cracks” – not detected until their health issues become serious and acute. Following the introduction of the respite service the level of acuity of admissions decreased which seemed to be a result of improved symptom management.

Aboriginal and Torres Strait Islander people comprised 76% of the participants, almost half of the participants were residents of town camps and 64% percent were suffering from chronic diseases. This highlights the complexity of the patient load in the respite service where individuals’ living situations are more likely to be poor and unstable, which is reflective of the NT population generally [1].

The respite facility filled a gap in services for those marginalised Aboriginal and Torres Strait Islander people suffering from chronic and complex conditions who are not yet in need of intensive end of life care. The participants were often isolated, living alone, and not accessing services either available to them or appropriately. Often symptoms were poorly managed and they had poor quality of life. The necessity of flexibility and cultural appropriateness in end of life care was emphasised fifteen years ago, [11] but these important considerations are still not realised on a widespread basis. McGrath et al. [11] also found that there was a shortage of respite services for Aboriginal and Torres Strait Islander people and their carers and these services were desperately needed.

A key aspect of this analysis was the calculation of cost savings to the health department brought about through the provision of a primary health care service that helps to prevent avoidable admissions to acute services. Once the respite service had started there was an average saving of almost $2000.00 per episode for hospital admissions leading to a total saving over the 10-month period of approximately $519,570.00 which amounts to an annual saving of approximately $623,484.00.

The total cost of running the respite service in the 2013–2014 financial year was $413,854.32. This includes the cost of set up, personnel, operational, and running costs. In this period, the total cost of running the service was offset by the savings to the health department. Moreover, when considering the cost of the service compared to the savings it produces it is important to remain cognisant of a number of influencing factors including set up costs (these are not ongoing) and complex issues such as homelessness which significantly impact on ED usage. Homelessness, however, cannot be addressed by the current respite facility as it is only a day service.

The mean length of stay in hospital for people accessing the respite service decreased by approximately 50% and average ICU hours decreased from 133.2 to 2. Mean ventilator hours reduced from 67.3 to 0. Interestingly, there was an *increase* in ED attendances as well as an increase in the number of admissions only to ED. The increase in ED hours was surprising and unexpected. These patients, however, are in terminal decline and need increasing clinical support. It may be that, through the care they received at the respite facility, they became less tolerant of problems they had previously accepted and sought assistance at an earlier stage. Thus, they sought increased services from ED, however, with better symptom management they required less admissions to hospital wards. A major finding of this study seems to be that these patients were much less likely to be admitted to acute care wards.

There was a slight reduction in the average number of admissions to the ward with almost a halving of the average number of days spent in hospital. These findings add further support to the suggestion of the benefits of improved symptom management. When the results of this study are considered in conjunction with the findings from the qualitative study [9] the overall impression appears to be that the support provided by the respite service resulted in an improvement in disease management by the provision of primary health care support, including support with medications, wound care, daily nutrition, and social and emotional support. The literature strongly indicates that a significant proportion of the deaths and hospital admissions in the NT are avoidable [12].

### Limitations

The study has limitations which affect the extent to which the findings can be generalised. The sample of patients were not randomly drawn from the target population so the generalisability of these findings is unclear. Furthermore, a pre- and post- design without a comparison group does not account for confounding factors. Remote palliative and chronic disease patients, however, are a specialised population, so it may be that the principles of ensuring that services are flexible, responsive, and culturally attuned will be important regardless of the locale to which these results are applied.

The time frame of this study was short because of budgetary and logistical constraints. A longer time period for both pre and post data would have increased the sample size and allowed firmer conclusions about the temporal stability of the changes to be made. Given the small sample size and the highly skewed distribution of the data, conducting tests of statistical significance was not considered appropriate or meaningful. The atypical nature of the data was well illustrated by the case studies described in the qualitative study [9].s With a larger sample size and longer timeframe, however, tests of statistical significance would be highly appropriate and would allow more robust conclusions to be made. It is not clear, however, as to the extent to which a longer timeframe would have altered the central message of this study which was that the day respite facility had a positive impact on the mean length of stay for people in hospital as well as the time spent in ICU and the number of ventilator hours used.

### Future research

It will be important in future research to extend the timeframe of the study. An extended timeframe would allow an increase in sample size and the ability to establish greater confidence in the financial benefits of the facility to the hospital. With the ongoing provision of the respite facility it may also be possible to investigate the effects of extensions of the service such as the option of overnight accommodation.

## Conclusions

The establishment of the respite service has addressed an important unmet need in Alice Springs; the provision of a respite service where none has existed before. The service assisted with cost savings to the health department and the funds saved could be used to contribute to the costs of the respite facility. Further cost savings from preventable hospitalisations can contribute to the ongoing and future costs of the respite facility. Establishing a flexible and responsive day respite facility for palliative and chronic disease patients in remote Australia with staff who understand the importance of being culturally attuned to their patients appears to have important implications for the rate of hospital admissions and subsequent costs to the local health service.
